# Commentary: Obstructive sleep apnea in the hemodialysis population: are clinicians putting existing scientific evidence into practice?

**DOI:** 10.3389/fneph.2024.1450204

**Published:** 2024-11-07

**Authors:** Aleena Jamal, Som P. Singh, Fawad Qureshi

**Affiliations:** ^1^ Sidney Kimmel Medical College, Philadelphia, PA, United States; ^2^ University of Missouri Kansas City School of Medicine, Kansas, MO, United States; ^3^ Department of Nephrology and Hypertension, Mayo Clinic Alix School of Medicine, Rochester, MN, United States

**Keywords:** obstructive sleep apnea, dialysis, referral, quality improvement, commentary

## Background

Obstructive sleep apnea (OSA) is well associated with an increased risk for various comorbidities and events, including cardiovascular disease and stroke (1). This condition is believed to affect nearly 1 billion individuals worldwide, and there is a growing concern for an underdiagnosis of OSA worldwide (2). There is also a body of evidence that describes a nuanced relationship between OSA and individuals with renal disease (3–6). Some studies have observed greater difficulty in diagnosing or identifying high-risk for OSA in patients with renal disease by using standard screening questionnaires, including the Adjusted Neck Circumference (ANC), Berlin Questionnaire, and STOP-BANG questionnaire, compared to the general population (7). Patients with renal disease who are appropriately diagnosed with OSA can allow clinical providers to better personalize management plans for their patients. For example, there is evidence that suggests patients with sleep apnea and chronic renal failure may be able to better correct their sleep apnea by undergoing nocturnal hemodialysis (8). Secondly, individuals with an appropriate and timely diagnosis of OSA can benefit from establishing a sleep plan with their OSA providers, including acquiring positive airway pressure devices (2, 9). This makes it critical for clinical providers to utilize strong judgment and leverage the tools necessary to diagnose and manage OSA in patients with renal disease. Moreover, this relationship was further explored in the article by Burkhalter et al. (4), evaluating diagnostic awareness of OSA by the patient’s nephrology providers. This commentary article aims to build upon the results found by this study.

## Discussion

A multicenter, cross-sectional study was described in the article by Burkhalter et al. (4), which aimed to explore further the relationship between OSA and end-stage renal disease patients undergoing hemodialysis and their nephrology providers. It assessed the prevalence of confirmed OSA diagnosis in this patient population and the awareness of this diagnosis among the nephrology providers. Another aim of the study was to analyze whether the knowledge of OSA adjusted the hemodialysis treatment plan that the nephrology provider offered to the patient. Regarding the prevalence, the investigators of this study found that 40% of the patients with a confirmed diagnosis of OSA were not correctly identified by the nephrology provider. Regarding the latter point, it was reported that only half of the nephrology providers reported awareness regarding “the pathophysiological link between fluid overload and OSA” and 25% reported using this knowledge in their patient’s hemodialysis fluid management plan (4). Among the salient findings in this study by Burkhalter et al., its data is suggestive that nephrology providers had difficulty in discerning OSA in their hemodialysis patients. It can be argued that the expertise of OSA truly is held by clinicians with formal training in pulmonary and sleep medicine. However, this makes it imperative for a multidisciplinary approach by nephrologists and primary medicine providers to refer their patients to OSA specialists. This commentary aims to suggest a possible quality improvement screening protocol for patients undergoing hemodialysis to better identify individuals at high risk for OSA. Two primary aims of OSA screening at a hemodialysis center would be to improve the underdiagnosis of OSA among patients with end-stage renal disease and to improve sleep education and quality. For patients on hemodialysis therapy and their caregivers, it is reported that sleep and fatigue/energy as ranked among the top priorities for hemodialysis outcomes rather than morbidity itself (10). This finding emphasizes the inherent value of this patient population in gaining quality sleep.

**Figure 1:**
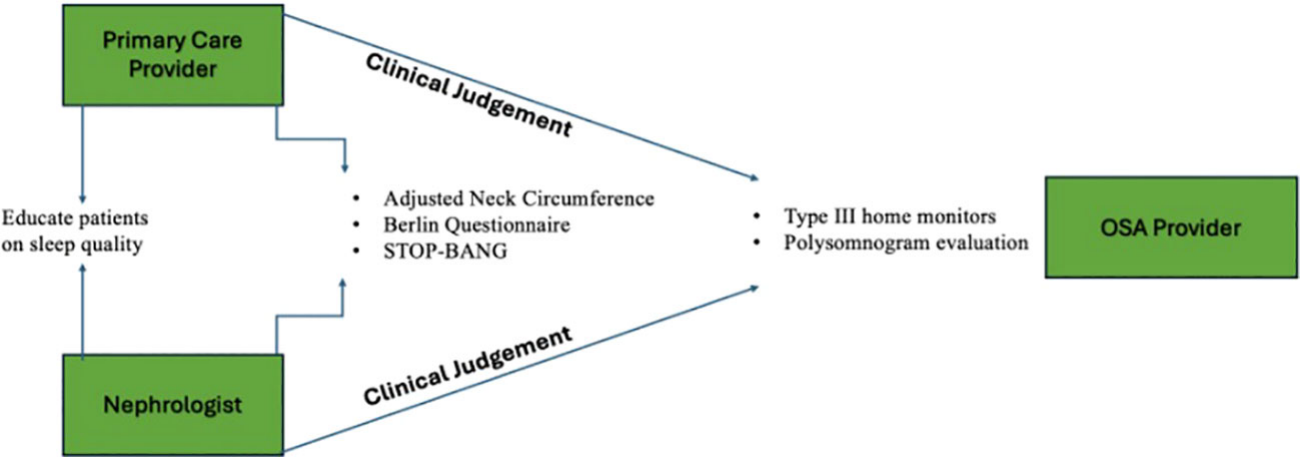
Multidisciplinary model for primary care physicians and nephrologists to utilize standardized OSA risk questionnaires and their clinical judgment to order instrumental tools to reduce the underdiagnosis of OSA in individuals undergoing hemodialysis. It is likewise essential for both provider groups to educate patients on the importance of quality sleep.

The multidisciplinary approach for a screening protocol for patients undergoing hemodialysis is demonstrated in **Figure 1** and can involve the primary care provider and the nephrologist. During follow-up visits, the patient’s primary care provider can utilize standard screening questionnaires (i.e., ANC, Berlin Questionnaire, STOP-BANG). While the current literature suggests that these questionnaires have demonstrated lower reporting rates for patients with end-stage renal disease and chronic kidney disease, they provide a cost-effective, rapid protocol that can be implemented. Current literature suggests that survey screening can be improved at the primary care level (11). Instrumental monitoring equipment, including type III home monitors and polysomnogram evaluations, would be superior in this patient population and potentially ergonomically advantageous given the nature of longitudinal monitoring during dialysis sessions compared to follow-up visits for a primary care provider (12). However, implementation costs could serve as a barrier to these methodologies (13, 14). On the other hand, a study also proposed and validated a diagnostic algorithm that could be implemented in the electronic medical record during dialysis sessions (15). This diagnostic algorithm utilized age (cutoff value: >70 years), neck circumference (cutoff value: >40 cm), and time of renal replacement therapy (cutoff value: >5 years). The study demonstrated that this algorithm performed better than standardized questionnaires for OSA risk stratification (15). However, there needs to be more data that investigates the proposal of a cut-off value of the number of criteria that need to be met to enter the subsequent level of screening proposed in this algorithm (i.e., ambulatory polygraphy). Moreover, the present limitations in the diagnostic algorithm include the age criteria which make it difficult to apply this algorithm in a generalized sense for individuals with ESRD under the age of 70. Additionally, most clinicians ought to consider that the weight loss associated with CKD diagnosis will reduce likelihood of referral for sleep apnea evaluation based on BMI criteria alone as well. Given that there is no guideline for appropriate screening of hemodialysis patients for OSA risk, the direction towards optimizing OSA screening in hemodialysis centers may rely upon increasing the awareness of nephrologists regarding signs of OSA in their dialysis patients and using their clinical judgment to implement instrumental sleep monitoring tests to confirm diagnosis. This suggests that dialysis centers should strengthen their sleep medicine infrastructure by providing one-time polysomnograms to patients enrolling in hemodialysis at the center or home sleep testing devices (16). Likewise, nephrologists should be able to educate patients regarding sleep hygiene and empower patients to monitor their sleep quality (17). This can empower patients to request a formal sleep evaluation from their nephrologist (18). Overall, these findings suggest the need for closer collaboration between primary care providers, nephrologists, and sleep medicine providers and the need to develop and validate personalized screening models for OSA for hemodialysis patients.
